# Oleoresin yield and carbon stocks in tapped subtropical *Pinus elliottii* forests

**DOI:** 10.1186/1753-6561-5-S7-P100

**Published:** 2011-09-13

**Authors:** Kelly Cristine da Silva Rodrigues-Corrêa, Tanise Luisa Sausen, Fernando S Rocha, Arthur Germano Fett-Neto

**Affiliations:** 1 Departament of Botany, Federal University of Rio Grande do Sul, Porto Alegre, RS, Brazil; 2 Center for Biotechnology, Federal University of Rio Grande do Sul, P.O. Box 15005, 91501-970 Porto Alegre, RS, Brazil; 3 Department of Botany and Center for Biotechnology, Federal University of Rio Grande do Sul, P.O. Box 15005, 91501-970 Porto Alegre, RS, Brazil

## Background

Low-cost methods to mitigate the increasing levels of carbon dioxide in the atmosphere and their implications on global climate change have received considerable attention in the last years [1,2]. Afforestation is an important alternative to reduce the rise in atmospheric CO_2_ concentration due to the system's ability to fix carbon in forest biomass and soil [3]. Several studies have been performed to estimate carbon sequestration in temperate, tropical, and mediterranean forests ecosystems [4,5]. However, there are no reports related to carbon balance in pine forests used for oleoresin tapping grown under subtropical climate. The main goal of this research is to estimate the effect of resin tapping on C sequestration by *Pinus elliottii* forests.

## Material and methods

### Resin tapping operation

In June 2009, 90 slash pine (*Pinus elliottii* Engelm.) trees of a 14 year-old forest were selected based on a previously determined DBH interval (between 23.48 ±1.12 and 22.77 ±0.88). Since the beginning of the essay, pine trees have been biweekly stimulated to produce oleoresin [6]. Three treatments were evaluated for pine biomass increase and oleoresin yield: bark streak (mechanical wound), paste (mechanical wound + chemical stimulation) and control (intact trees). At the end of each season, the released oleoresin was collected and weighed in a digital balance. The results shown below were obtained between Spring 2009 and Winter 2010.

### Biomass production and carbon accumulation

In November 2010, fifteen pine trees (5 intact tree control, 5 mechanically wounded, and 5 paste stimulated tapped trees) were felled and weighed in the field to estimate the above and belowground total fresh biomass of trees. Sub-samples of each part of trees were collected and dried in an oven at 105°C until reaching constant dry weight. The carbon content per unit dry weight present in the aboveground and belowground biomass was estimated using a carbon content of 50% [7]. Data were analyzed for differences between resin treatments (bark streak vs. paste) by comparison of means by ANOVA and Tukey test (Systat Software Inc., Richmond, CA USA); significance was set at P *£*0.05).

## Results and conclusions

**Figure 1:**
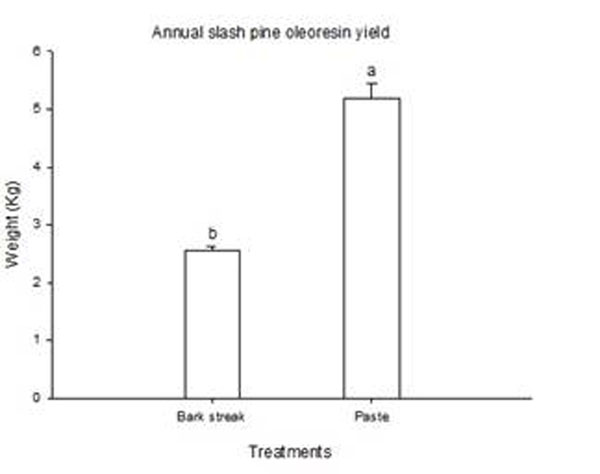
Annual production of oleoresin by pine trees. Standard errors of the means are indicated on top of bars. Bars with different letters indicate significant difference by a Tukey test (*P*≤ 0.05). Each mean was calculated with 30 individual trees.

The seasonal oleoresin production did not show the same pattern previously observed [8]. The highest oleoresin yield was observed in Spring (data not shown). Statistical differences between oleoresin yields of paste-treated and control (bark streak) trees were observed (Figure 1). These results are in agreement with the fact that besides genetic traits, physiological status, season and environmental conditions [9-11], inducible oleoresin biosynthesis consistently responds to exogenous chemical stimuli [6,8].

**Figure 2:**
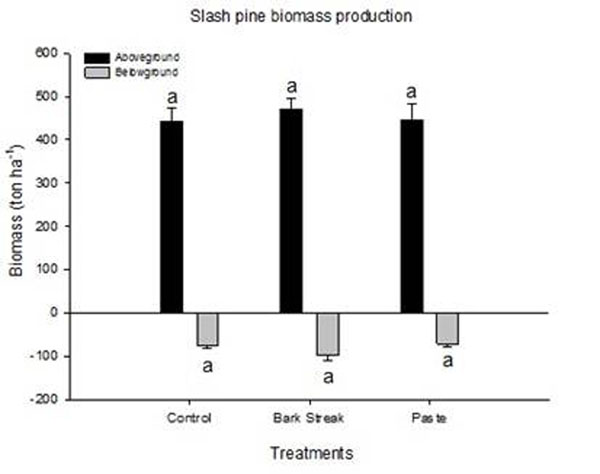
Aboveground and belowground biomass among the treatments (control, bark streak and paste). Standard errors of the means are indicated on top of bars. Each mean was calculated with five individual trees. Identical letters indicate no significant difference by a Tukey test (*P* ≤0.05).

The production of aboveground biomass expressed on a dry weight basis was significantly higher than that of belowground biomass. This was observed for all treatments, and no significant differences were detected in biomass production and partition for tapped (both plain wound and chemical stimulation) or control trees (Figure 2). The aboveground biomass showed a carbon accumulation between 221.3 and 235.9 Mg C ha^-1^ and in the roots, the carbon accumulation is in the range between 35.7 and 48.3 Mg C ha^-1^.

These results indicate that carbon stored in aboveground biomass appeared to represent the main carbon pool of tree biomass and that resin tapping had minor impact on C allocation in wood biomass, considering the time frame and stand age examined. Further studies and sampling times are ongoing in order to better characterize carbon stocks in subtropical slash pine plantations and to elucidate the contribution of oleoresin production in carbon stocks and its relation with C in wood biomass.
